# Neutrophil-like cells derived from the HL-60 cell-line as a genetically-tractable model for neutrophil degranulation

**DOI:** 10.1371/journal.pone.0297758

**Published:** 2024-02-07

**Authors:** Suhani B. Bhakta, Stefan M. Lundgren, Bethany N. Sesti, Barbara A. Flores, Emel Akdogan, Sean R. Collins, Frances Mercer

**Affiliations:** 1 Department of Biological Sciences, California State Polytechnic University Pomona, Pomona, CA, United States of America; 2 Department of Microbiology and Molecular Genetics, University of California Davis, Davis, CA, United States of America; Jahangirnagar University, BANGLADESH

## Abstract

Research on neutrophil biology has been limited by the short life span and limited genetic manipulability of these cells, driving the need for representative and efficient model cell lines. The promyelocytic cell line HL-60 and its subline PLB-985 can be differentiated into neutrophil-like cells (NLCs) and have been used to study neutrophil functions including chemotaxis, phagocytosis, endocytosis, and degranulation. Compared to neutrophils derived from hematopoietic stem cells, NLCs serve as a cost-effective neutrophil model. NLCs derived from both HL-60 and PLB-985 cells have been shown to perform degranulation, an important neutrophil function. However, no study has directly compared the two lines as models for degranulation including their release of different types of mobilizable organelles. Furthermore, Nutridoma, a commercially available supplement, has recently been shown to improve the chemotaxis, phagocytosis, and oxidative burst abilities of NLCs derived from promyelocytic cells, however it is unknown whether this reagent also improves the degranulation ability of NLCs. Here, we show that NLCs derived from both HL-60 and PLB-985 cells are capable of degranulating, with each showing markers for the release of multiple types of secretory organelles, including primary granules. We also show that differentiating HL-60 cells using Nutridoma does not enhance their degranulation activity over NLCs differentiated using Dimethyl Sulfoxide (DMSO) plus Granulocyte-colony stimulating factor (G-CSF). Finally, we show that promyelocytic cells can be genetically engineered and differentiated using these methods, to yield NLCs with a defect in degranulation. Our results indicate that both cell lines serve as effective models for investigating the mechanisms of neutrophil degranulation, which can advance our understanding of the roles of neutrophils in inflammation and immunity.

## Introduction

Neutrophils are essential first-responder cells in most immune responses to pathogens, and at sites of injury or damage. They are the most abundant leukocyte in human blood, and rapidly extravasate into infection sites in large numbers to attack pathogens through multiple effector mechanisms, including phagocytosis, trogocytosis, oxidative burst, casting of neutrophil extracellular traps (NET-osis), and exocytosis of toxic granules (degranulation) [1, 2]. Degranulation is a particularly important effector function and a defining feature of neutrophils, which are characterized in part by their distinctive granules. When neutrophils are activated, the toxic granules are exocytosed, releasing a range of microbicidal products including proteases, lipases, and reactive chemical species, thus mediating pathogen attack and tissue damage, as well as amplifying inflammation [3–5]. The toxic granules also play roles in several of the other effector mechanisms. For example, toxic granules can fuse with a maturing phagolysosome, to aid in degradation of internalized pathogens [4] and granule contents can both aid in the induction of NETs [6], and co-localize with extracellular NETs, serving to damage ensnared pathogens within [3].

Neutrophil toxic granules come in multiple types: primary (azurophilic), secondary (specific), tertiary (gelatinase), and quaternary (secretory vesicles), with a fifth type (Ficolin-1-rich granules) also recently identified [4]. Each granule has specific cargo and transmembrane proteins. The four classic neutrophil granule types are named in order of their appearance during granulopoiesis, with primary granules developed first, and secretory vesicles not appearing until neutrophils are terminally differentiated. However, upon stimulation, the granules are released in reverse order, which corresponds with content toxicity. Primary granules, which are the most toxic, require potent stimulus and are released last. Meanwhile, secretory vesicles contain minimal toxic components and are the most poised for release [4].

How degranulation is selectively employed in response to particular threats, and the extent of the overlap between the mechanisms of release of different granule types is not fully understood, particularly in the human system. These questions are important for human health. Identifying strategies to limit degranulation in response to particular stimuli, or to limit the release of granules by subtype, could allow the development of targeted anti-inflammatory drugs with reduced side effects. The use of model systems will be critical for resolving these mechanisms.

While mouse models offer genetic tractability, mouse genetics are slow and resource-intensive. Furthermore, murine and human neutrophils have important differences, including differences in granule contents [7, 8], in their signaling pathways following activation [9], and in the chemokines they respond to [10]. Finally, mechanistic studies of neutrophil interactions with human-restricted pathogens can often not be done with mouse neutrophils. Studies of neutrophil functions in humans have been hampered by the short life span of the cells ex vivo: primary neutrophils only survive for about a day following isolation from the peripheral blood, severely limiting the amount of forward and reverse genetics that can be done to identify regulatory players in human neutrophil functions. Therefore, researchers have sought creative strategies to model human neutrophils for in vitro experiments, such as differentiating neutrophils in vitro from CD34+ hematopoietic stem cells (HSCs) [11–13], or from induced human pluripotent stems cells (iPSCs) [14, 15]. With proper signals, HSCs can follow the differentiation process into terminally-differentiated neutrophils with mature neutrophil effector functions. In this process, HSCs first differentiate into myeloid progenitors, then into myeloblasts, and then to committed neutrophil progenitors, which begin the process of granulopoiesis to acquire their toxic granules. Granulopoiesis continues during further sequential differentiation into promyelocytes, myelocytes, metamyelocytes, and finally into terminally-differentiated neutrophils [4, 6, 16]. Thus, using HSCs is technically intensive.

The promyelocytic leukemia cell-lines HL-60 and PLB-985 offer an attractive alternative to HSCs, because they are further along in the differentiation pathway, and can be differentiated in vitro to become neutrophil-like cells. Furthermore, they grow robustly in conventional tissue-culture media, eliminating the need for expensive stem-cell growth factors needed for differentiating neutrophils from HSCs. Therefore, researchers have used neutrophil-like cells (NLCs) differentiated from the HL-60 promyelocytic cell line, or its subline PLB-985, as a model to study various neutrophil functions including phagocytosis, chemotaxis, oxidative burst, and NETosis [17–19]. While researchers have used NLCs derived from both HL-60 and PLB-985 cells for studies of degranulation [20–29] no studies have directly compared which cell line model and conditions are optimal for these studies. Furthermore, their potential for genetic investigation of degranulation is still largely unexplored.

Here, we compare the ability of NLCs derived from either HL-60 or PLB-985 cells to degranulate. We analyze primary granule exocytosis, as well as markers for other secretory organelles, and we examine both a classical and newer differentiation protocol to generate the NLCs. We find that NLCs derived from both cell lines display degranulation activity, with NLCs derived from HL-60s showing higher activity, although the difference between the cell lines was not statistically significant. We also detected the highest amount of degranulation from HL-60 cells differentiated using classical methods. We find that both cell lines secrete primary granules, secretory vesicles, and CD107a-marked secretory organelles. Finally, using CRISPRi, we generated a promyelocytic cell line that can be differentiated into NLCs with impaired degranulation, showing the genetic tractability of this model. Our results establish both cell lines as suitable models for studies on the mechanisms and impacts of human neutrophil degranulation.

## Materials and methods

### Cell culture

HL-60 or PLB-985 cells were cultured in complete RPMI, which contained HEPES, Glutamax, penicillin- streptomycin, and 10% fetal bovine serum (all from GIBCO and used according to the manufacturer’s instructions) at 37°C in 5% CO_2_ and passaged at least every 3 days to maintain cells at 2 x 10^5^ to 1.8 x 10^6^ cells/mL.

### Differentiation to neutrophil-like cells (NLCs)

HL-60 or PLB-985 cells were differentiated to become “neutrophil-like” in by plating cells at 2 x 10^5^ cells/ml in complete RPMI (as described above) supplemented with 1.3% Hybri-max DMSO (Sigma-Aldrich), and 100 ng/mL carrier- free Granulocyte Colony Stimulating Factor (Biolegend), or in complete RPMI supplemented with 1.3% Hybri-max DMSO (Sigma-Aldrich) and 2% Nutridoma-CS (Sigma). Cells were then left undisturbed for 6–7 days at 37°C in 5% CO^2^.

### Stimulating NLCs

NLCs were plated for stimulation at 2 x 10^5^ NLCs in 200ul of complete RPMI (as described above) in a u-bottom 96-well plate and were incubated with either 100ng/mL PMA (Calbiochem) for 15 minutes (PMA), or with 10uM cytochalasin B (ThermoFisher) for 5 minutes followed by 5uM fMLP (fMLP) for 15 minutes (Sigma Aldrich) at 37°C in 5% CO_2_.

### Confirmation of NLC degranulation

Immediately following the stimulation protocols above, supernatants were gently removed and stored at -20°C. Then, the cells were immediately fixed with 4% paraformaldehyde and incubated for 15 minutes at 37°C before incubating with 1ug/mL of FITC anti-human CD63, FITC anti-human CD35, or APC anti-human CD107a antibody or isotype control (Biolegend) for 30 minutes at 4°C. Cells were then run through either a FACS Calibur (Becton-Dickenson) or a MACS Quant 10 (Miltenyi Biotech) flow cytometer and analyzed using FlowJo (Becton-Dickenson). The isotype control showed a lower signal at all conditions than the anti-CD63, anti-CD35, and anti CD107a antibody conditions. For detection of myeloperoxidase (MPO), supernatants stored overnight or several days were thawed slowly on ice, and tested using the BioLegend LEGENDplex^TM^ Human MPO Capture Bead kit according to the manufacturer’s instructions. Samples were then run through either a FACS Calibur (Becton-Dickenson) or a MACS Quant 10 (Miltenyi Biotech) flow cytometer and analyzed using FlowJo (Becton-Dickenson).

### Rab27a knockdown

Knockdown cell-lines were generated using two rounds of 2nd generation lentiviral-mediated gene transfer. First, a cell-line expressing a dCas9-KRAB fusion was created. This cell-line was used in a second round of infection to stably express sgRNA complimentary to the Rab27a promoter. The lentiviral packaging vector used was a gift from Dr. Lifeng Xu (pMD.G, University of California, Davis). The envelop vector was a gift from Dr. Peter Lewis (pCMV-dR8.2, University of Wisconsin, Madison). HEK-293T cells were transfected in Opti-MEM (Gibco, catalog # 51985–034) using TransIT-2020 transfection reagent (VWR, catalog # 10767–014). Media was changed to DMEM containing 10% FBS after 12 hours. Supernatant was collected at 24 and 48 hours post media change and filtered (PES 0.45μm; catalog # 25–246). Lentivirus containing supernatant was concentrated 1:50 as described by the manufacturer (Origene, catalog # TR30026), flash frozen in liquid nitrogen and stored at -80°C. PLB-985 cells were treated with 50 μl of the concentrated lentivirus and 2 mg/ml Polybrene (Sigma; catalog # H9268-5G). 18 hours later, transduced cells were centrifuged (100G; 10 minutes) and resuspended in selection media (supplemented RPMI-1640).

RAB27A expression was quantified in the CRISPRi knock-down cells using Real-Time PCR (RT-PCR). RNA was extracted using Qiagen RNeasy RNA extraction kit according to the manufacturer’s protocol, from differentiated cells on 3 different days (biological replicates). 1000 ng of purified messenger RNA (mRNA) was reverse transcribed to complementary DNA (cDNA) using the 5x All-In-One RT Master Mix kit (abm). Approximately 20 ng cDNA template was used in the RT- PCR reaction (KiCqStart® SYBR® Green qPCR ReadyMix™) with 3 technical replicates for each sample. We used the LightCycler® 480 Instrument with the following conditions: 95°C for 5 s, 45 cycles of 95°C for 10 s, 55°C for 15 s, 72°C for 20 s, melting curve of 95°C for 5 s, 65°C for 1 m, cooling. We designed primers that span exon-exon junctions and the primers used were as follows: For RAB27A, forward: 5’-gggcgagccagacaaaaag-3’, reverse: 5’-acagggtagagaaccgcttg-3’, and for G6PD (reference), forward: 5’-gtgacctggccaagaagaag-3’, reverse: 5’-gaagggctcactctgtttgc-3’. To quantify enrichment in RAB27A expression in guide expressing cells relative to dCas9-KRAB cells, we used the delta-delta Ct (ΔΔCt) method.

### Statistical analysis

Where indicated, statistical significance was determined using a one or two-tailed student’s T-test, or three-way ANOVA as indicated in the figure legends, where * indicates p<0.05, ** indicates p<0.01, and *** indicates p <0.001.

## Results

**Fig 1:**
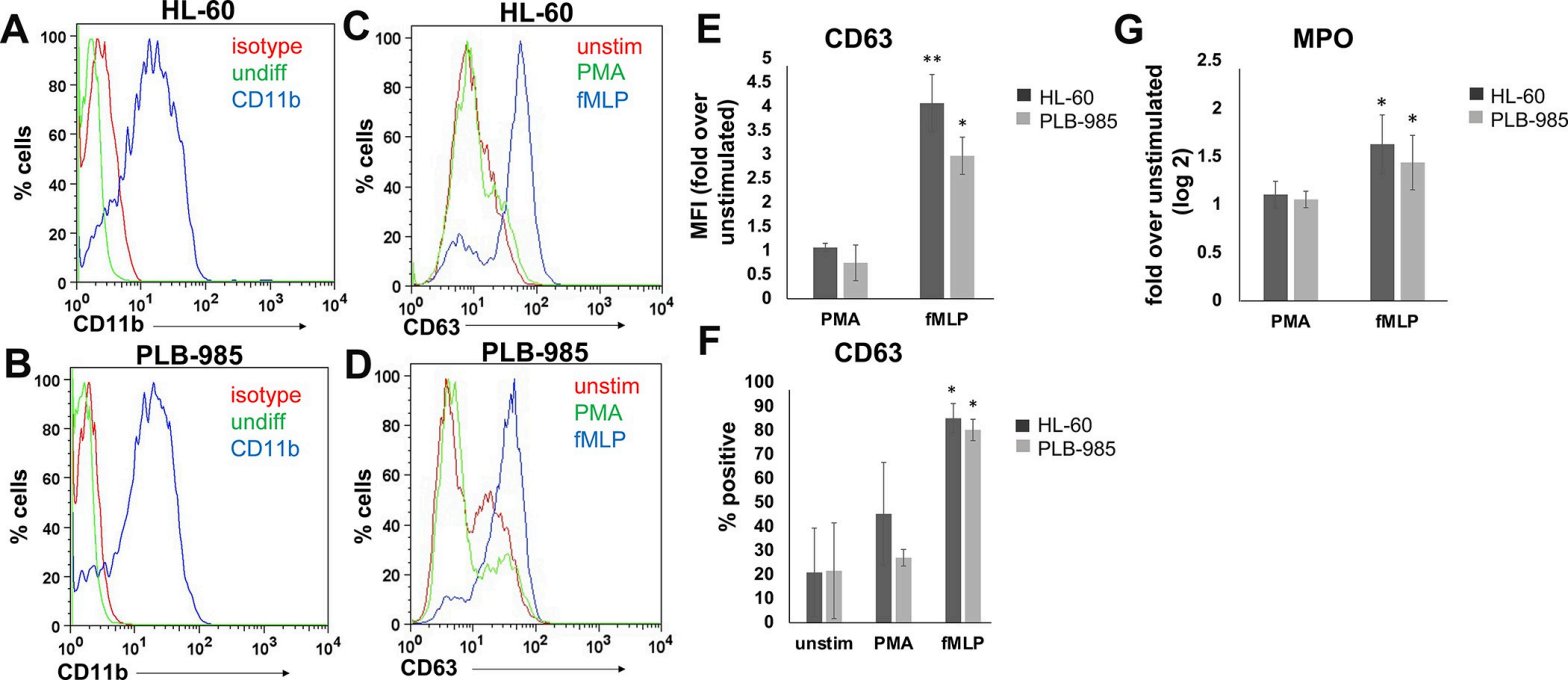
Neutrophil-like cells derived from HL-60s and PLB-985 cells show exocytosis of primary granules. HL-60 or PLB-985 cells were differentiated into Neutrophil-like cells (NLCs) and tested for exocytosis of primary granules. (A, B) NLCs were stained for differentiation marker CD11b, or an isotype-matched control. CD11b levels are compared to undifferentiated cells as an additional negative control. Data are representative of three experiments. (C, D) NLCs were differentiated and then left unstimulated (unstim), or stimulated with either PMA, or cytochalasin B and fMLP (fMLP). Then, cells were stained with anti-CD63 antibody (C-F) and supernatants were tested for secretion of MPO (G). Panels C and D show representative histograms for one experiment performed in triplicate, and E-G are the average of three independent experiments, each with 3 biological replicates. Error bars show the standard deviation and statistical comparison to unstimulated controls was done using a one-tailed students t-test. There was no statistically significant difference between the stimulated conditions for any panel.

The promyelocytic cell lines HL-60 and PLB-985 are established cell lines that can be differentiated into neutrophil-like cells (NLCs), with CD11b serving as a reliable marker for differentiation [19, 27]. After confirming differentiation of the cells (Fig 1A and 1B), we began comparing degranulation conditions for differentiated NLCs. We first selected markers to efficiently assess degranulation. While neutrophils contain multiple types of toxic granules that can be released during a degranulation event, primary granules require the strongest stimuli to induce exocytosis and therefore are the last granules to be exocytosed following a degranulation trigger [4]. We selected two complementary indicators to use as proxies for full neutrophil degranulation, one of which appears on the cell surface and a second which is secreted. As a surface marker, we used CD63, which is localized to primary granule membranes and exhibits increased cell surface levels upon granule exocytosis [30, 31]. For a second indicator, we measured myeloperoxidase (MPO) secretion into the supernatant [30]. MPO is an enzyme that diversifies reactive oxygen species production and is contained only within the primary granule subtype [30, 31].

To determine which promyelocytic cell line could differentiate into NLCs with greater degranulation activity, we differentiated HL-60 and PLB-985 cells into NLCs using traditional methods [19]. We then stimulated the NLCs and measured their ability to increase CD63 surface expression and secrete MPO into the supernatant (Fig 1C–1G). To induce degranulation, we used cytochalasin B followed by N-Formyl-Met-Leu-Phe (fMLP) [28]. We compared the degranulation activity in our stimulated NLCs to unstimulated NLCs, or to NLCs activated with phorbol 12-myristate 13-acetate (PMA), which can activate neutrophils to produce reactive oxygen species (ROS), but does not activate granule exocytosis [32]. We found that NLCs only degranulated when stimulated with fMLP, and did not degranulate when stimulated with PMA, or when left unstimulated (Fig 1C–1G). Furthermore, we found that NLCs derived from both cell lines showed degranulation activity (Fig 1C–1G). While HL-60 cells showed consistently greater degranulation activity than the PLB-985 subline, this difference was not statistically significant (Fig 1C–1G). Also, while a majority of the NLCs derived from both cell lines did show substantially increased surface localization of CD63 upon stimulation (Fig 1C and 1D), the percentage of cells with surface-expressed CD63 did not reach 100% (Fig 1F), suggesting heterogeneity for degranulation capabilities within the cell lines. This could be due to a small fraction of cells that did not fully differentiate (Fig 1A and 1B).

**Fig 2:**
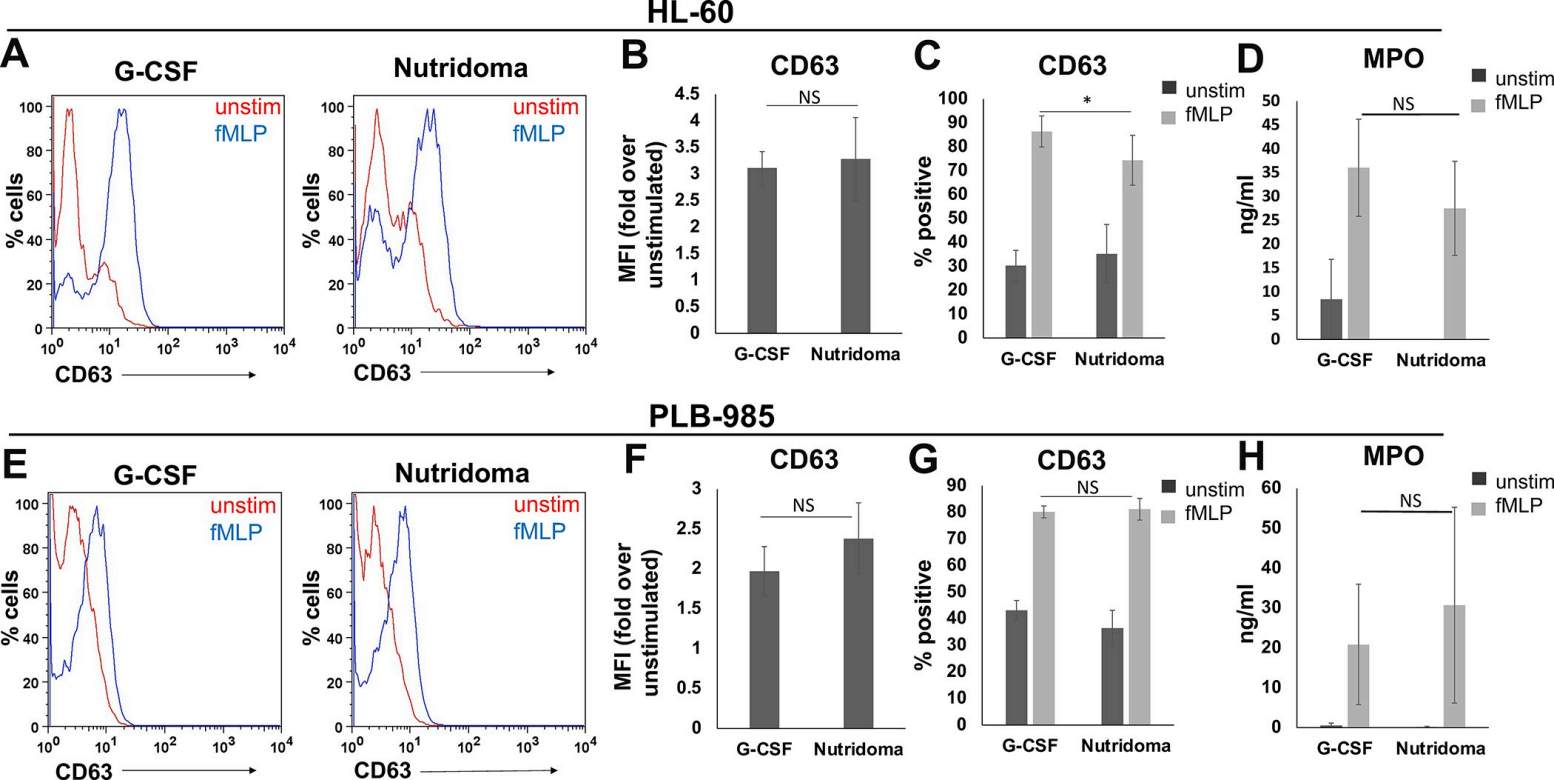
Nutridoma differentiation of promyelocytic cells does not substantially improve degranulation ability. HL-60 (A-D) or PLB-985 (E-H) cells were differentiated into Neutrophil-like cells (NLCs) with DMSO, in addition to either G-CSF or Nutridoma and then left unstimulated (unstim), or stimulated with cytochalasin B and fMLP (fMLP). Then, cells were stained with anti-CD63 antibody (A-C, E-G) and supernatants were tested for secretion of MPO (D, H). Panels A and E show representative histograms for one experiment performed in triplicate and B-D, F-H are averages of three independent experiments. Error bars show the standard deviation and statistical comparison between treatments was done using a two-tailed student’s t-test.

We next sought to determine whether degranulation activity of NLCs could be improved by differentiating the cells with Nutridoma, a proprietary cocktail historically used in hybridoma culture, which has also been used to enhance differentiation of promyelocytic cells in some instances [19, 27]. Recently, differentiation of promyelocytic cells with Nutridoma was shown to increase the resultant NLCs’ ability to undergo phagocytosis, oxidative burst, and chemotaxis [19]. Therefore, we hypothesized that differentiation of promyelocytic cells with Nutridoma would increase the resultant NLCs’ ability to undergo degranulation as well. To test this, we differentiated HL-60 or PLB-985 cells with either a conventional method, utilizing DMSO + Granulocyte-Colony-Stimulating Factor (GCSF), or using the newer Nutridoma method of DMSO + Nutridoma. Then, we stimulated the resultant NLCs and tested their ability to degranulate. We found that Nutridoma did not improve the ability of NLCs differentiated from HL-60s to degranulate (Fig 2A–2D). While we did see a modest increase in degranulation of NLCs derived from PLB-985 cells after differentiation with Nutridoma, (Fig 2E–2H), this increase did not reach statistical significance. The modest increase may be due to heterogeneity in the expression of the formyl peptide receptor FPR1 in differentiated PLB-985 cells, which is reduced with the Nutridoma protocol [19]. However, overall levels of degranulation from the PLB-985 subline were still lower than degranulation from NLCs derived from HL-60 cells with either differentiation protocol (Fig 2).

**Fig 3:**
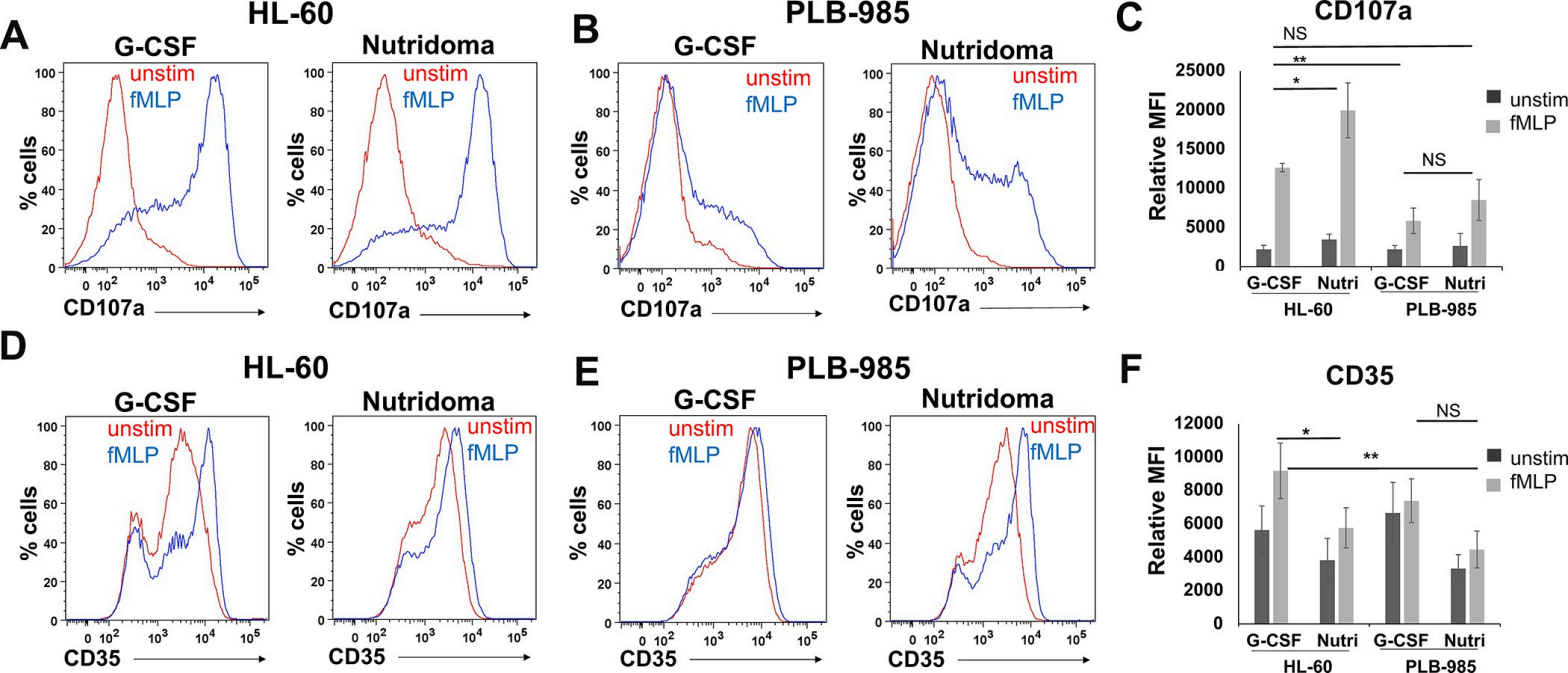
Neutrophil-like cells derived from promyelocyte precursors release other mobilizable organelles including secretory vesicles. HL-60 or PLB-985 cells were differentiated into Neutrophil-like cells (NLCs) with DMSO, plus either G-CSF or Nutridoma and then left unstimulated (unstim) or stimulated with cytochalasin B and fMLP (fMLP). Then, cells were stained with anti-CD107a (A-C) or anti-CD35 (D-F) antibody. Panels A, B, D and E show representative histograms of triplicate samples, and C and F are averages of three independent experiments. Error bars show the standard deviation and statistical comparison between the samples was performed using a three-way ANOVA, after confirming normality and equal variance using the Shapiro-Wilk test and Levene’s test respectively. The stimulated conditions were found to be significant compared to their unstimulated controls for all cell lines and differentiation methods tested, with a p-value of 1 x 10^−4^ and 1 x 10^−6^ for Fig 3C and 3F respectively. Two-tailed T-tests between comparisons of interest are also shown.

Considering that neutrophils release multiple granule types and secretory organelles, we also sought to determine if NLCs were a capable model more generally for these secretion events by measuring additional markers including CD107a and CD35. CD107a, or LAMP1, is reported to mark mobilizable organelles including multivesicular bodies, but not primary granules, in neutrophils [33, 34]. We found that the NLCs do exocytose CD107a to the cell surface in response to fMLP (Fig 3A–3C). CD35 is a well-established marker of secretory vesicles [35, 36]. While it has partial basal localization to the plasma membrane, its abundance at the membrane increases upon secretion of these vesicles [37]. We also observed CD35 exocytosis to the cell surface, indicating that secretory vesicles were released, and supporting that our NLCs were terminally differentiated, as these are the last granules to develop during granulopoiesis [4, 30].

**Fig 4:**
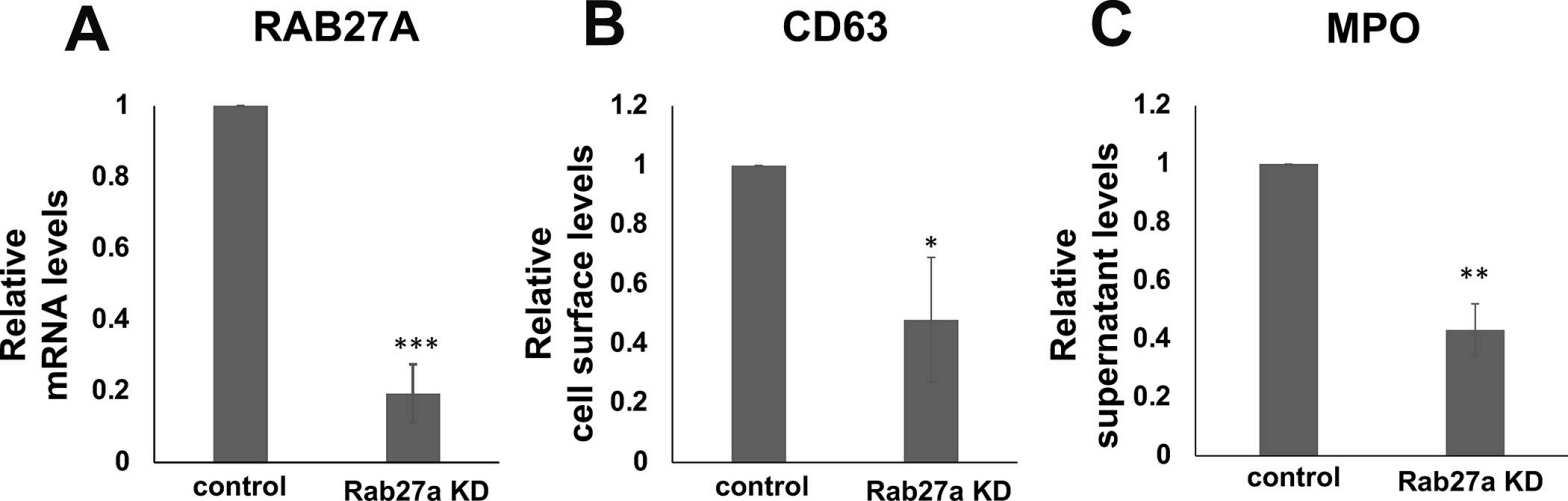
Genetically altered promyelocytic precursors yield Neutrophil-like cells with impaired degranulation functions. (A) Bar chart shows the average relative RAB27A expression levels calculated using the delta-delta Ct (ΔΔCt) method and normalized to a G6PD control. (B) Measurement of primary granule exocytosis by increased CD63 membrane expression in differentiated PLB-985 cells expressing dCas9-KRAB (control) compared to PLB-985 cells expressing dCas9-KRAB and Rab27a sgRNA. (C) Measurement of degranulation activity through MPO release in differentiated PLB-985 cells expressing dCas9-KRAB (control) compared to PLB-985 cells expressing dCas9-KRAB and Rab27a sgRNA. For each panel, the data are averages of three independent experiments performed in triplicate. Error bars represent the standard deviation and statistical comparison was done using a two-tailed student’s t-test.

After confirming degranulation capabilities in NLCs, we sought to determine if promyelocytic cells can be used as a genetically-tractable system to test neutrophil granule functions. Therefore, we designed a CRISPRi strategy to knock down Rab27a, a Rab family small GTPase that is critical for mobilization of primary granules from the cytosol to the plasma membrane where they can dock and fuse to release their contents. Previous studies show that siRNA-based interference of Rab27a expression impairs primary granule release [38, 39]. Here, we sought to determine viability of CRISPR based strategies by verifying that knockdown of Rab27a would result in impaired degranulation. We chose PLB-985 cells for these experiments, as they grow more robustly in culture and have higher viability following lentiviral transduction. Following introduction of CRISPRi constructs and confirmation that Rab27a transcript levels were reduced (Fig 4A), we differentiated cells using DMSO and G-CSF, and then treated them with Cytochalasin B and fMLP to induce degranulation. We measured plasma membrane levels of CD63, and MPO release. We found that the Rab27a knockdown cells had significantly reduced degranulation capabilities, verifying the usefulness of NLCs as a genetically tractable model for degranulation (Fig 4B and 4C).

## Discussion

Neutrophils have myriad effector mechanisms, with the mobilization of toxic granules being among the most essential to both their antimicrobial and inflammatory functions [1]. However, studies of how degranulation is regulated, and its full impact, have been hampered by the short-lived nature of the terminally differentiated cells, and the high cost of differentiating primary cells in vitro [31, 40]. Promyelocytic cell lines therefore offer an attractive option for in vitro studies of neutrophil degranulation [19]. The goal of the work herein was to establish a genetically-tractable model of human neutrophil degranulation, in order to enable future studies to investigate molecular players in neutrophil degranulation and to understand the impacts of neutrophil degranulation on other cellular processes. Here, we found that both HL-60s and their subline, PLB-985 cells can be differentiated to NLCs that display degranulation activity, and that promyelocytic cells offer a genetically tractable model system for studies of degranulation.

Researchers have identified various markers of neutrophil degranulation present on NLCs derived from HL-60 and PLB-985 cells in response to various stimuli over the past 40 years. For example, NLCs derived from PLB-985 cells have been shown to exocytose primary granules, tertiary granules, and secretory vesicles [27–29], and NLCs derived from HL-60s have been shown to exocytose primary granules [20–25] and tertiary granules [22, 25, 26]. Thus, our studies are in alignment with previous reports because they show that NLCs derived from both PLB-985 and HL-60 cells can exocytose primary granules. However, past studies used different differentiation strategies, stimuli, and granule markers, making it difficult to directly compare the degranulation activity of NLCs derived from one cell line over the other.

In our studies, we sought to identify key markers that could most succinctly assess degranulation and use these as a standard to compare the degranulation activity of NLCs derived from HL-60s to those derived from PLB-985 cells, as well as to measure any reduction in degranulation activity achieved by knockdown of a gene involved in degranulation. As primary granules are the last granules to be released during a degranulation event and require the strongest stimulus to induce [4], we decided that primary granule exocytosis was a suitable marker for full degranulation. We chose CD63 and MPO in order to measure cell-surface changes associated with primary granule exocytosis, as well as a secreted primary granule content. After finding that NLCs derived from HL-60 cells and the PLB-985 subline showed primary granule exocytosis, we also confirmed that HL-60-derived NLCs cells were terminally differentiated, because they show increases in the surface expression of a secretory granule marker (secretory granules develop last during granulopoiesis) upon stimulation [4], as well as CD107a, which is present on other mobilizable organelles including multivesicular bodies in neutrophils [33]. Therefore, our results suggest that promyelocytic cells differentiated with G-CSF and DMSO, have completed granulopoiesis, and can fully degranulate upon stimulation with fMLP. Eosinophil-like cells differentiated from HL-60 cells have also been used as a model to study degranulation in eosinophils [32, 41–43], lending further support to the utility of the HL-60 promyelocytic cell line in studies of granulocyte degranulation.

NLCs derived from HL-60 cells and differentiated and stimulated in the same way that we do here, have also been shown to exocytose tertiary granules [22, 26]. However, there are no reports confirming that NLCs derived from either HL-60 cells or PLB-985 cells can exocytose secondary granules, and some studies have suggested that secondary granules fail to develop in promyelocytic cell line models [22, 44]. However, the paradigm of discrete, well-defined granule types has been challenged, and granules may exist as more of a spectrum [4, 35, 45]. In this vein, we note that the CD107a marker, which is correlated with granule release, is a useful indicator of degranulation. Furthermore, because of its low surface expression prior to stimulus, it has a high signal-to-noise ratio compared to other markers that we tested.

Some researchers are now also examining the degranulation activity of neutrophils derived from HSCs [11]. Secondary granules have been identified in neutrophils derived from stem cell precursors [15]. However in another study, while primary granules were found to be intact and functional, secondary granules appeared abnormal when compared to primary neutrophils [6]. Therefore, stem-cell derived neutrophils, while costlier and more time consuming to prepare, may still not fully recapitulate primary neutrophil granulopoiesis and granule function.

Another limitation to the use of promyelocytic cell lines is possible inconsistency from line-to-line in different laboratories. PLB-985 cells and HL-60 cells are old and commonly used laboratory cell lines. Therefore, the source of the cells may vary from lab-to-lab, and culture bottlenecks may produce variability or artifacts. Indeed, the PLB-985 line may have come from cross-contamination with HL-60 cells, followed by slight functional divergence over time [46]. Here, we used HL-60 and PLB-985 cell lines that were extensively characterized for neutrophil functions, and for which extensive RNA-seq analyses were done on both the promyelocytic, and fully differentiated NLCs, which are publicly available [19].

Taking the above considerations into account, we therefore conclude that promyelocytic cells lines, differentiated with DMSO and G-CSF, and stimulated with fMLP are a useful model for studies of human neutrophil degranulation. In particular, we propose CD63 and CD107a as useful screening markers for degranulation. The genetic tractability of this model, as demonstrated by stable RAB27A knockdown using CRISPRi further demonstrates its utility for loss-of-function experiments to probe questions about the regulation and impacts of human neutrophil degranulation.

Neutrophils are of crucial importance in immune responses, as their abundance in the blood, their swift and numerous appearance at inflamed sites, and the immediacy by which they attack targets upon contact enables them to mediate essential roles in killing pathogens and limiting their dissemination. However, these same properties are associated with driving tissue damage in many inflammatory and autoimmune pathologies [47–49]. The mechanisms they use to mediate these effects are numerous and complex, representing some of the most interesting cell-biological functions occurring in mammalian cells. Once thought to function like suicide bombs, reacting with all of their effector mechanisms at once and dying in the process, both the short-lived and inflammatory nature of all neutrophils [50, 51], as well as their indiscriminate reaction to stimuli are being challenged [52]. The use of genetically tractable systems to assess human neutrophil biology will therefore potentiate a better understanding of this crucial player in health and disease, potentiating the design of more effective interventions for both infectious and autoimmune diseases.

## Supporting information

S1 Data (XLSX)
